# ‘Challenges in pediatric medicine access in Albania: insights from community Pharmacists’

**DOI:** 10.1080/20523211.2025.2521425

**Published:** 2025-07-02

**Authors:** E. Petro, T. Martopullo, A. K. Mantel-Teeuwisse, H. A. van den Ham, F. Suleman

**Affiliations:** a Primary Healthcare Services, Local Healthcare Unit, Durres, Albania; b WHO Collaborating Centre for Pharmaceutical Policy and Regulation, Division of Pharmacoepidemiology and Clinical Pharmacology, Utrecht Institute for Pharmaceutical Sciences (UIPS), Utrecht University, Utrecht, the Netherlands; c Lady of Good Counsel University, Tirana, Albania; d WHO Collaborating Centre for Pharmaceutical Policy and Evidence Based Practice, Discipline of Pharmaceutical Sciences, School of Health Sciences, University of KwaZulu-Natal, Durban, South Africa

**Keywords:** pediatric medicines, access, pharmacist perception, barriers, facilitators, Albania

## Abstract

**Background:**

Medicines are crucial for strengthening health systems and building patient confidence. Yet, there is a global shortage of age-appropriate pediatric formulations, particularly in low- and middle-income countries. Pharmacists are vital in ensuring pediatric medicines’ availability and proper use. Research has often overlooked pediatric needs, resulting in significant gaps in understanding the accessibility of medicines for children. This research therefore sought to identify the barriers and facilitators to accessing pediatric medicines from the perspective of community pharmacists in Albania.

**Methods:**

Semi-structured interviews were conducted in March 2024 with 18 members of the Albanian Order of Pharmacists, working in the six most populated cities in Albania. Interviews were transcribed verbatim and analyzed using the Pharmaceutical Value Chain framework.

**Results:**

Barriers to accessing pediatric medicines were evident in almost all domains of the Pharmaceutical Value Chain framework except for health information technology, a domain not referred to by participants. Issues addressed included a limited number of items on the reimbursement list for different child-specific diagnoses and occasional stock-outs of child-appropriate medicines. Open communication channels with other healthcare professionals and patient education were identified as key facilitators in improving access to pediatric medicines.

**Conclusion:**

This is the first study to examine pediatric medicine access in Albania from the viewpoint of community pharmacists. The findings provide context-specific insights that can inform policy reforms and health system strategies to improve the equitable availability of pediatric medicines.

## Background

Access to medicines is a fundamental pillar of effective health systems worldwide, essential for ensuring quality healthcare delivery and achieving positive health outcomes (World Health Organization, n.d.a). However, the availability of age-appropriate formulations of pediatric medicines remains a significant challenge, particularly in low- and middle-income countries (LMICs) (Ainscough et al., 2018; Best et al., 2011; Orubu & Tuleu, 2017; Richey et al., 2017; Zahn et al., 2020). This issue is critical given that children under 5 years of age in LMICs face high mortality rates due to preventable diseases and inadequate treatment options (UNICEF, 2024). Addressing this gap is essential for improving child health outcomes and reducing mortality.

The World Health Organization (WHO) underscores the vital importance of making pediatric medications accessible as a key element in strengthening health systems, emphasising their crucial role in enhancing child health worldwide (World Health Organization, 2019). Research efforts have traditionally focused on measuring access to medicines for the general population, without particular consideration for medicines for children. As a result, there is a major gap in our understanding regarding accessibility of medicines for children (Joosse et al., 2022). Efforts to address these challenges have highlighted the multifaceted nature of barriers faced by healthcare providers, and pharmacists in particular, who play a critical role in ensuring the availability and appropriate use of medicines (Sakeena et al., 2018 & Smith & Olin, 2010).

Pharmacists serve as key stakeholders in the pharmaceutical value chain (PVC), bridging the gap between healthcare providers and patients. They are instrumental in optimising medication access and management, especially for pediatric populations. They play a crucial role in ensuring that pediatric patients receive appropriate medications and dosages, addressing the unique challenges of pediatric care to improve treatment outcomes (Wolters, n.d.). Global research highlights disparities in access to essential pediatric medicines, with notable gaps in LMICs where infrastructure and funding constraints hinder effective distribution and availability (Yenet et al., 2023). Researchers in Belgium and Canada have explored medicine shortages and the critical role of pharmacists in managing them. Although their studies do not specifically target pediatric medicines, literature indicates that shortages are more pronounced for these treatments. In Europe, pharmacists face regulatory and logistical challenges, while in Canada, they must navigate complex national and international supply chains to address these shortages (De Weerdt et al., 2017; Panic et al., 2020). Despite efforts to improve access, there remains a significant lack of comprehensive studies exploring how pediatric medication accessibility is specifically addressed within different national healthcare systems (Cohen et al., 2018; Ernest et al., 2007; Mwangi et al., 2022).

Albania continues to face systemic challenges in ensuring equitable access to pediatric medicines, despite having a relatively high density of pharmacists (Oculus, 2019). Key challenges include limited domestic pharmaceutical production, a strong dependence on imported medicines, and the lack of targeted policies to support the registration and steady supply of pediatric formulations (UK Department for Business and Trade, 2023).

Albania’s medicine supply relies heavily on imports, with domestic pharmaceutical production accounting for only around 10% of the market and primarily focused on generic adult formulations. Pediatric-specific medicines are rarely produced locally, increasing reliance on international manufacturers and creating persistent availability gaps (UK Department for Business and Trade, 2023). Furthermore, the national reimbursement list managed by the Health Insurance Fund is only partially aligned with global standards. A recent comparative analysis found that just 48.2% of shared active ingredients between Albania’s reimbursement list and the WHO Essential Medicines List for Children (EMLc) were matched in dosage form, strength, and indication (Petro et al., 2023). This misalignment contributes to limited access to age-appropriate formulations for children. While Albanian pharmacists are trained according to national professional and ethical standards (Urdhëri i Farmacistëve të Shqipërisë, n.d.), there is limited evidence on how these competencies are applied in the context of pediatric medication access. In particular, little is known about how frontline pharmacists perceive and navigate systemic issues such as medicine shortages, pricing challenges, and the lack of age-appropriate formulations.

Alongside this, child health issues persist, including high rates of preventable diseases and gaps in treatment access. In 2022, the under-5 mortality rate was reported at 9 per 1,000 live births, higher than the European average of 4 per 1,000, highlighting the potential for further improvement (UNICEF, n.d.).

Addressing these gaps and disparities is crucial for improving child health outcomes, particularly given the country's evolving healthcare landscape. Albania’s healthcare system is undergoing significant transformation, marked by decentralisation, investments in primary healthcare, and efforts to align national health policies with European standards. These reforms aim to improve service delivery and financial protection, yet systemic inefficiencies and disparities in access to care – particularly for children – persist (World Health Organization, 2018). Albania operates a mandatory health insurance scheme managed by the Health Insurance Fund, covering a defined list of services and medicines. However, the system provides partial financial protection with out-of-pocket (OOP) health expenditure that was at or above 50% of current health expenditure since 2000, reaching 51.3% in 2022 (World Health Organization, n.d.b). This high reliance on OOP payments poses a significant burden for households, particularly in accessing medicines. This requires targeted research efforts to understand current practices in facilitating pediatric medication access in Albania and what challenges are encountered from the perspective of the pharmacist. Such studies are crucial for informing evidence-based interventions that can enhance medication availability and optimise healthcare outcomes for children.

### Aim

This study aims to examine the perspectives of community pharmacists regarding access to pediatric medicines in Albania, with the objective of identifying real-world barriers and facilitators to generate context-specific evidence in order to inform equitable and responsive pharmaceutical policy interventions.

## Methods

### Study design

A qualitative approach was adopted to explore community pharmacists’ perspectives on pediatric medicines access, as they serve as key stakeholders in the pharmaceutical value chain and are directly involved in the daily navigation of medicine shortages, caregiver interactions, and reimbursement constraints. This approach is appropriate for exploring system-level challenges from a frontline perspective and complements prior qualitative work in access to medicines research (Boateng et al., 2021; Joosse et al., 2024).

### Sampling of participants

This qualitative component was part of a broader cross-sectional study conducted using the WHO/HAI methodology for facility selection. Within this framework, purposive sampling was used to recruit experienced community pharmacists from Albania’s six most populated cities, Tirana, Durrës, Kavajë, Lushnjë, Fier, and Vlorë. to gather insights into medicine access In December 2023, the Albanian Order of Pharmacists invited 24 licensed pharmacists (four per city) via email or text message. All invitees were active members of the Order and regularly involved in dispensing pediatric medicines.

Eighteen pharmacists agreed to participate. The sample ensured both geographic spread and professional relevance. The number of interviews was also guided by the principle of data saturation. After about 15 interviews, no new important themes emerged, and the last few interviews helped confirm and enrich the results.

### Interview guide

A semi-structured interview guide was developed based on existing literature (Carroll et al., 2023; Institute of Medicine (US), 2008; Santer et al., 2014; Spadoni, 2019; WHO, 2010; WHO, n.d.c; Young et al., 2002) and included 15 open-ended questions. These questions focused on four main areas: pharmacists’ experiences with pediatric medicine dispensing, adherence to clinical guidelines, financial aspects, and the effects of medicine access on treatment adherence. Prompts were used to encourage detailed and reflective responses. The guide was pilot-tested with two pharmacists not included in the final sample, and minor changes were made to improve clarity and flow.

Although the interview guide was not developed using a formal framework, the analysis was structured deductively using the Pharmaceutical Value Chain (PVC) framework (Joosse et al., 2024). The PVC is a systems-based model that outlines key processes in pharmaceutical access, including regulation, pricing and financing, selection, procurement and supply, delivery, dispensing, use, and health information systems. It allows for the identification of barriers and facilitators at multiple points in the medicine access pathway.

Interview data were first grouped under relevant PVC domains. Within each domain, inductive coding was applied to identify sub-themes and patterns emerging from participant responses. This combined deductive – inductive approach enabled both structured analysis and the exploration of new insights based on pharmacists’ lived experiences.

The full interview guide is available in Appendix 1 in the online Supplemental Material.

### Data collection

Before the interviews, participants were briefed on the research objectives and topics by EP (female), a PhD candidate, and TM (male), a medical student. Both interviewers had prior experience in conducting interviews, ensuring their proficiency with the methodological approach used. The researchers confirmed that there were no pre-existing relationships with the participants, which helped maintain impartiality. This introductory phase was intended to establish transparency and clarify the study’s purpose. While the recorded interview segments ranged from 4 to 19 min, these interviews were conducted as part of a broader access to pediatric medicines research study and additional observational data were also collected (not audio recorded) during these visits. All interviews followed a structured guide with appropriate probing by trained interviewers. Participants received the guide in advance, allowing for prior reflection and preparation (to collate and condense their response) due to their limited time in their busy facility. In some cases, participants declined to answer specific questions, because they felt the question did not directly relate to their professional role. Despite these variations, data saturation was reached, with recurring themes evident across the transcripts. All interviews were conducted within a single week in March 2024 by EP and TM at the participants’ workplaces. Written and verbal consent was obtained from all participants before recording began. During the interviews, only the researchers and participants were present, and the interviews were conducted in Albanian to ensure natural and comprehensive responses.

Detailed field notes were recorded during the interviews to capture contextual nuances and emotional undertones, complementing the verbatim audio recordings. Following verbatim transcription and translation into English, a subset of four randomly selected transcripts was returned to participants for review. This review process aimed to validate transcription accuracy, confirm that the transcriptions faithfully represented participants’ responses, and provide an opportunity for participants to clarify any discrepancies or provide additional context, without altering their original statements. This approach enhanced the reliability of data interpretation by ensuring that the transcripts accurately reflected the content and intent of the interviews.

### Data analysis

The authors evaluated data saturation to ensure comprehensive thematic exploration. Data analysis was guided by the Pharmaceutical Value Chain (PVC) framework, a comprehensive systems-based model covering ten interconnected domains that affect medicine access: policy and legislation, regulation, selection, public financing and pricing, reimbursement, procurement and supply, healthcare delivery, dispensing, use, and health information systems (Joosse et al., 2024). This framework was selected for its suitability in qualitatively analyzing access to medicines from a health systems perspective.

A three-order coding process was used to organise the data:

- First-order coding captured direct statements and descriptive codes from transcripts.
- Second-order coding grouped these into sub-themes reflecting patterns across interviews.
- Third-order coding involved mapping sub-themes onto the ten PVC domains, enabling structured interpretation across the medicine access pathway.

Initial coding of six transcripts was collaboratively done by EP and FS to ensure consistency. The remaining interviews were coded independently by the first author. The analysis combined inductive coding (to allow new themes to emerge) and deductive coding, using the structure of the PVC framework. Sub-themes were then summarised to highlight key insights relevant to pediatric medicine access in Albania.

This dual approach facilitated a comprehensive analysis. Data interpretation involved summarising each sub-theme after identifying similarities and differences. Confidentiality of participants was maintained throughout the study, and data storage adhered to legal requirements, including compliance with the EU General Data Protection Regulation (GDPR).

### Ethics approval and consent

This study involved human participants and received ethics approval from the Science-Geosciences Ethics Review Board (SG ERB) of Utrecht University (Approval No. Sci R-23.011, dated 7 February 2024) and the Albanian Order of Pharmacists (Protocol No. 948, dated 11 December 2023). All participants received written and verbal information about the study objectives and procedures. Informed consent was obtained prior to participation, and each participant signed a consent form. Participation was voluntary, and participants had the right to withdraw from the study at any time without consequence.

## Results

Eighteen pharmacists agreed to participate and be interviewed, while six declined due to a preference not to be recorded. The final sample included 14 female and 4 male participants, with an average of 18.6 years of professional experience. A detailed overview of participant characteristics, including age, gender, experience, and city, is provided in Appendix 3 in the online Supplemental Material.

A total of 19 sub-themes were identified through thematic analysis, mapped under nine of the ten processes outlined in the Pharmaceutical Value Chain (PVC) framework. Each PVC domain represents a third-order theme, with associated second-order categories and first-order participant insights. The full coding tree is presented in Appendix 2 in the online Supplemental Material.

### Policy and legislation

In Albania, the pricing and regulation of medicines significantly affect healthcare accessibility and quality. Community pharmacists discussed national pharmaceutical policies in general terms, particularly those related to pricing reforms and standardisation of active ingredients. However, no specific legislation or policy measures focusing exclusively on pediatric formulations or pediatric pharmaceutical regulation were mentioned. The observations in this domain therefore reflect broader pharmaceutical governance issues that may also influence pediatric medicine availability Pharmacists have raised concerns about a policy aimed at standardising active pharmaceutical ingredients and supplements to match regional pricing structures. They believe this decision, made by the Council of Ministers, could impact the quality of medicines by reducing the variety of available brands. There are worries that such standardisation could limit pharmaceutical diversity and potentially decrease overall quality (see Table 1).

**Table 1.** Selected quotes for the themes: policy and legislation & medicine regulation.

**Policy and legislation**
*– ‘I think that the difference of the active principles molecules and supplements was caused by the decision of the Council of Ministers, because they requested the unification with the prices of the region, and the active principles were already referring to the prices of the region.’ (R15)*
*– ‘For this aspect, I have an issue, because this leads to a decrease in quality and at the same time limits the arrival of brands.’ (R15)*
**Medicine regulation**
*– ‘I try to keep everything. I try to keep the medicines that circulate and are in the pharmaceutical yearbook of the Republic of Albania. They [the medicines found in the pharmacy] try to respond to the brand that the doctor wants.’ (R18)*
*– ‘The liberalization of the market caused the work in pharmacies to decline and, to the withdrawal of more medicines than necessary, and it is difficult to quantify of what needs to change. But the availability of pediatric medicines is currently lower than previous consumption levels.’ (R18)*
*– ‘There are also medicines that are not registered, there are even essential medicines that are not registered that are missing from the market. Some have been registered in the past but are no longer available, while others have never been.’ (R3)*

### Medicine regulation

Participants highlighted various challenges and impacts within the medicine regulation framework. Community pharmacists prioritise stocking medications listed in the reimbursed list by the Mandatory Health Insurance Fund (yearbook) in pharmacies. This adherence to the reimbursed list ensures compliance with health insurance policies and regulations. Pharmacists expressed concerns about the liberalisation of the market, which broadly affected medicine availability. However, participants specifically noted a decline in the availability of pediatric medicines, due to reduced profitability and stock prioritisation. Increased competition and economic pressures might lead pharmacies to stock fewer pediatric medications, as they may prioritise more profitable or higher-demand products. This market shift has also resulted in an increase in unnecessary medicine withdrawals and a shortage of essential medicines, including some that are either no longer registered or have never been available, highlighting significant gaps in meeting pediatric healthcare needs (see Table 1).

### Public financing and pricing

Pharmacists had differing views regarding the variability in price between adult and pediatric medicines. While some pharmacists noted no pricing difference in general, others emphasised that pediatric medicines are often slightly more expensive, especially those requiring specialised formulations like syrups and dispersible tablets. These higher costs were cited as a barrier uniquely affecting families seeking care for children. Participants emphasised the impact of high medication prices on parents and caregivers, who often make strategic prioritizations based on financial constraints when accessing pediatric medications. Parents sometimes request cheaper alternatives or change the active ingredient to afford necessary medications for their children. Additionally, there are cases where parents cannot afford certain medications due to their high cost, leading them to prioritise the most urgent needs. Despite these financial challenges, the sacrifices parents make for their children's health were highlighted as they generally prioritise their children's health over financial concerns (see Table 2).

**Table 2.** Selected quotes for the themes: public financing and pricing & selection.

**Public financing and pricing**
*– ‘It is the same for adults, it is not that there is any difference between children and adults in this case.’ (R9)*
*– ‘Yes, there is a difference. In general, pediatric medicines tend to be priced slightly higher than adult medicines.’ (R14)*
*– ‘Yes, it has an impact. There are even cases where they tell us to find something cheaper, which may lead to changing the active ingredient, just so that they can afford to buy that type of antibiotic for their children.’ (R7)*
*– ‘There are medicines that they cannot take because they are more expensive than they can afford.’ (R17)*
*– ‘Yes, there are certain classes that [parents] can't afford … . then [they] say what is the most urgent to get, what can I leave for now.’ (R2)*
*– ‘No, parents generally make sacrifices for their child, and price is not a controversial issue for them,’ (R5).*
**Selection**
*– ‘In general, yes, they [protocols] are understandable and more than easily accessible. We have not encountered any difficulties in this aspect.’ (R9)*
*– ‘Unfortunately, we have limited access to approved clinical protocols and, in this regard, lack sufficient information about patient profiles and diagnoses. Despite private efforts, this lack of detailed information makes it challenging to assess or understand the prescriptions made by doctors.’ (R17)*
*– ‘No, we don't have any [protocols], but the medicine lists are written according to the protocol and we learn from experience.’ (R18)*
*– ‘We know very well that a quinolone preparation, for example fluoroquinolone, is not used in pediatrics.’ (R18)*

### Selection

Regarding treatment guidelines, pharmacists expressed diverse perspectives on their accessibility and utilisation within Albania's healthcare system. Some found treatment guidelines to be generally accessible and understandable, while others reported challenges in accessing approved clinical protocols and lacking detailed information about patient profiles and diagnoses. In practice, pharmacists often rely on experience and informal knowledge gained from everyday work and peer discussions. This reliance underscores the importance of pharmacist training and knowledge, particularly in avoiding certain medications in pediatric cases (see Table 2).

### Reimbursement

Participants pointed out significant deficiencies in the coverage and availability of pediatric medicines listed for reimbursement, expressing concerns about the insufficiency of current options. They highlighted constraints in reimbursement, particularly in accessing essential medications such as antibiotics, noting that pediatric patients benefit minimally from the current reimbursement list. Observations revealed discrepancies between prescribed pediatric medications and those eligible for insurance coverage, with some previously reimbursed prescriptions now categorised differently. Participants advocated for policy improvements to expand the reimbursement list to better meet pediatric healthcare needs (see Table 3).

**Table 3.** Selected quotes for themes: reimbursement & procurement and supply.

**Reimbursement**
*– ‘The range of pediatric medications covered in the reimbursable medicine list is typically very limited’ (R17)*
*– ‘Pediatric patients benefit very minimally from the current reimbursement list.’ (R15)*
*– ‘Previously, there were prescriptions with reimbursements, but now they are all categorized as R3 prescriptions.’ (R12)*
*– ‘There is a need to advocate for legislative changes to enrich the reimbursement list with pediatric medications.’ (R15)*
**Procurement and supply**
*– ‘They are generally generic. Rarely can there be a patent medicine, but most of the ones we give out during the day are all generic.’ (R7)*
*– ‘At certain moments and periods, there are shortages in the market.’ (R14)*
*– ‘There are generally more medicines for adults.’ (R5)*
*– ‘Patients don't like replacements. If there are shortages, we refer them to the doctor to see how it can be replaced.’ (R2)*

# R3 prescriptions refer to medications that are not covered by reimbursement schemes. This means that patients are responsible for paying the full cost of these medications out-of-pocket.

### Procurement and supply

Pharmacists observed that supply chain challenges impacted medicine availability broadly; however, they frequently emphasised that the range of available pediatric medicines is notably narrower than that for adults. While adult medications were generally stocked in larger quantities and greater variety, essential pediatric formulations, such as salbutamol inhalers and amoxicillin syrups, were often in short supply or completely absent. These represent pediatric-specific supply constraints that directly hinder access to care for children (see Table 3).

### Healthcare delivery

Participants highlighted challenges in implementing the referral system for pediatric prescriptions in Albania, noting that parents often bypass this system by directly consulting pediatric specialists. This can lead to the avoidance of reimbursement protocols, as medications are only reimbursed if the patient first visits the specialist through a referral from a general practitioner, which is frequently circumvented in cases of acute conditions.

Integration and coordination of healthcare services were emphasised as crucial for enhancing operational efficiency and maintaining smooth prescription handling across different healthcare settings. A well-connected system would ensure that all aspects of patient care, from hospital visits to pharmacy dispensing, are part of a closed cycle of service control and re-control, providing a clearer picture of service efficiency.

The generation of pediatric prescriptions involves ensuring that the prescribing doctor meets professional qualifications and adhering to procedural steps to maintain prescription integrity and appropriateness. Participants observed a preference for prescriptions written with trade names over generic names, which complicates medication selection based on factors such as patient financial resources. Enhancing the use of generic names could improve medication access, allowing pharmacists to better ensure that patients receive medications listed on the reimbursement list or the most cost-effective options available. Implementing a standardised electronic prescription system for reimbursed medicines could further optimise cost efficiency and access to necessary medications. *See* Table 4.

**Table 4.** Selected quotes for theme: healthcare delivery.

**Healthcare delivery**
– *‘ … who have an acute or chronic problem, but mostly acute, they don't go to the doctor, they don't follow the route of referrals but go directly to the specialist, so we bypass the reimbursement in this way, while those who have chronic problems, they do … .’ (R1)*
– *‘ … we should have a very connected system. If we were in the system, it would be known that the child is visited in the pediatric hospital of the city of Vlora, the medicines are taken in this pharmacy. It is a closed cycle not in financial terms, but a closed cycle of service control and again in re-control, and then we can investigate and have a clearer picture of their efficiency.’ (R15)*
*– ‘To generate a prescription, in principle, the doctor must be a specialist or maybe even a general practitioner, but the generation requires the implementation of several types of procedures.’ (R1)*
– *‘Usually, we have prescriptions that are written by trade names, we do not receive prescriptions that are named after the chemical component so that we can choose depending on the population, the financial recourses of the patient or many other factors. But they come to us written with commercial names, that is, according to an R3 prescription so that the doctor determines what the patient, a child in this case, will receive*.’ (R7)
– ‘*But my personal opinion is that we should work with generic names, it should be improved, there is a lot of room for improvement. Should another electronic prescription be made, such as for medicines on the reimbursement list, there is much room for improvement*.’ (R18)

### Dispensing

Pharmacy practices in Albania have evolved significantly, moving away from in-house compounding of medications to standardised dispensing practices. Pharmacists emphasised their responsibilities in ensuring prescription accuracy and patient safety, including calculating doses based on the child's weight if not specified in the prescription and consulting with doctors as needed. Collaboration with healthcare providers is crucial for resolving prescription ambiguities and enhancing patient care. When managing medication shortages and substitutions, pharmacists aim to substitute with the same active ingredient or consult the doctor for alternatives (see Table 5).

**Table 5.** Selected quotes for the theme: dispensing.

**Dispensing**
*– ‘When I first started, children's medicines like Pertussin syrup were prepared in pharmacies. Now, however, we no longer compound these types of medications.’ (R10)*
*– ‘We calculate the dose based on the child's weight if it's not specified in the prescription. We strictly adhere to the prescribed dose and consult the doctor if needed.’ (R2)*
*– ‘Yes, there is a difference in pediatric medicines and medicines for adults, you need to be very careful with the dose, because in children the dose calculation is based on weight.’ (R8)*
*– ‘We prefer direct communication or refer the patient back to the doctor if necessary.’ (R2)*
*– ‘When a medication isn't available, we substitute with the same active ingredient or consult the doctor for alternatives.’ (R16)*

### Use

Community pharmacists reported that medicine shortages affect both adult and pediatric populations; however, particular concern was expressed regarding the unavailability of essential pediatric treatments, such as salbutamol inhalers used for managing asthma in children. These shortages were perceived to have a greater impact on pediatric care due to the limited availability of suitable alternatives. Adherence to pediatric treatment regimens was also highlighted as a challenge. Pharmacists noted that children or their caregivers often discontinue medications, such as syrups for respiratory symptoms, once initial improvements are observed. This pattern of premature discontinuation was identified as a pediatric-specific adherence issue, emphasising the importance of sustained caregiver education and follow-up. While substitution with equivalent active ingredients is a common practice, it was reported to be more difficult in pediatric settings. Caregivers of children were often hesitant or unwilling to accept substitutes, even when they were therapeutically equivalent, due to concerns about safety, unfamiliarity, or perceived differences in efficacy. These barriers, rooted in communication gaps and trust, were seen as more prevalent in pediatric contexts than in adult care (see Table 6).

**Table 6.** Selected quotes for the theme: use.

**Use**
*– ‘One of the main problems currently is the breathing pump, i.e. salbutamol which is currently in short supply.’ (R7)*
*– ‘In cases, for example, when it is prescribed that cough syrup will be used, there are cases that [patients] stops as soon as they see an improvement, so there are cases that that advice is not followed until the end.’ (R2)*
*– ‘If there is an alternative at the molecule level, absolutely we [substitute] it.’ (R1)*
*– ‘The difficulty is only in the lack of understanding from the patient, i.e. the parent of the child who often does not understand, for example, that we are on the same active principle and refuses.’ (R7)*

## Discussion

### Key findings

This qualitative study, based on 18 interviews, elucidates the diverse perspectives of community pharmacists regarding the accessibility and availability of pediatric medications within the Albanian healthcare system. Key issues identified include challenges with accurate dosing, medication shortages, and the exclusion of essential pediatric medicines from the reimbursement scheme. Financial constraints for parents and market dynamics further exacerbate access barriers. Additionally, the study highlights the implications of standardisation policies on the quality and variety of pediatric pharmaceuticals. The study underscores the unique complexities in pediatric care, such as the need for precise dosing, age-appropriate formulations, and tailored adherence strategies, since children may need different approaches to ensure they take their medications as prescribed, considering factors like taste preferences, fear of needles, or the difficulty of swallowing pills.

While community pharmacists are not directly involved in regulatory decision-making or national policy design, their insights reflect operational constraints that emerge from upstream issues such as deregistration, weak reimbursement coverage, and the absence of pediatric-tailored incentives. These perspectives are essential for triangulating system-wide barriers to access and can inform future policy design by highlighting where caregiver needs and frontline service gaps intersect. Similar studies in LMICs have demonstrated the value of such insights in informing national pharmaceutical policy reform (Boateng et al., 2021; Joosse et al., 2024; Wirtz et al., 2017)

### Interpretation and implication

The findings from this study highlight critical issues impacting pharmaceutical practice and pediatric healthcare accessibility in Albania. Pharmacists expressed significant concerns regarding policy and regulatory decisions, particularly the standardisation of active pharmaceutical ingredients and supplements to align with regional pricing structures. This policy directive was perceived to potentially compromise medicine quality and restrict the variety of available brands. Similar concerns about the impact of regulatory measures on medication diversity and quality have been noted in previous studies in other countries (Conroy et al., 2000; Roughead et al., 2018).

These community pharmacist-reported challenges reflect broader structural issues in Albania’s pharmaceutical system. The country’s limited manufacturing capacity, combined with a strong reliance on imports, contributes to medicine shortages and limited pediatric options. Moreover, the current reimbursement list excludes several essential pediatric formulations. A recent analysis by Petro et al. (2023) found that fewer than half of the shared active ingredients between Albania’s national reimbursement list and the WHO Essential Medicines List for Children (EMLc) were fully aligned, underscoring systemic barriers to accessing child-appropriate treatments. These findings highlight the need for greater alignment of national policies with international standards and for incentives to support pediatric medicine registration and supply.

Participants also voiced concerns about the consequences of market liberalisation, highlighting a decrease in pharmacy activity and increased instances of medicine shortages, particularly for pediatric medications. These issues underscore the challenges imposed by market dynamics and regulatory frameworks in maintaining adequate pharmaceutical supplies for children. This situation is analogous to findings in research by Boateng et al. (2021), which examined access to childhood cancer medicines across four Caribbean countries. Their study identified similar barriers to medication access, including regulatory and market-related challenges, underscoring the widespread nature of these issues and reinforcing the relevance of their comparative analysis to understanding and addressing pediatric medication shortages (Boateng et al., 2021).

The financial challenges reported by community pharmacists align with broader national concerns, as Albania continues to experience high levels of out-of-pocket spending, especially for outpatient medicines. This weakens financial protection and may exacerbate inequalities in access, particularly for pediatric populations. Community pharmacists have observed that pediatric medications frequently come at a higher cost compared to adult medicines, which poses significant financial barriers for families. Studies have shown that the production and formulation of pediatric medications often require specialised processes and smaller batch sizes, which can drive up costs. Additionally, the comparative costs of treating children versus adults within certain diagnosis-related groups highlight the financial disparity, emphasising the economic strain placed on families (Young et al., 2002). In certain diagnosis-related groups, the cost of treating pediatric patients can be disproportionately high due to the need for specialised formulations (e.g. syrups, mini-tablets), age-appropriate packaging, and additional safety and dosing requirements. These formulations often involve higher per-unit production costs and smaller batch sizes, making them more expensive than adult equivalents and thereby increasing the financial burden on families – particularly in the absence of comprehensive reimbursement (World Health Organization, 2024).

Furthermore, the transition from in-house compounding to standardised dispensing practices has been identified as a major shift in pharmacy operations. This change is intended to improve accuracy and patient safety in medication administration. However, this shift toward standardised dispensing practices has also introduced new challenges in pediatric care, including reduced flexibility in dose preparation, limited ability to compound age-appropriate formulations, reliance on brand-name prescriptions, and persistent mismatches between prescribed medicines and those eligible for reimbursement. According to the International Pharmaceutical Federation (FIP), standardised practices are crucial for ensuring the reliability of pediatric medicine delivery and manufacturing. The FIP highlights that while these practices enhance consistency and safety, they also require careful consideration of the unique needs of pediatric patients to avoid compromising treatment efficacy (International Pharmaceutical Federation, n.d.).

The challenges of managing medication shortages and substitutions were also apparent in the study. Pharmacists detailed strategies such as substituting medications with identical active ingredients or consulting physicians for alternative treatments during supply disruptions. This flexibility is crucial for ensuring continuity of care despite market fluctuations and supply chain disruptions. Furthermore, Huss et al. (2023) highlighted that these shortages, particularly in pediatric medications, have become a significant issue across Europe, exacerbated by global dependencies and logistical problems. Their research emphasised the urgent need for improved coordination and systemic reforms to address these shortages effectively. In particular, their findings underscored the importance of proactive measures such as stock redistribution, alternative medicine use, and enhanced production strategies to mitigate the impact on pediatric care and ensure that essential medications remain accessible (Huss et al., 2023).

While no specific comments were provided regarding health information systems (HIT), the pharmacists’ role in coordinating care and improving prescription management is critical. HIT can enhance communication among pediatricians, pharmacists, and caregivers, ensuring everyone is on the same page regarding a child's treatment plan and any adjustments needed (Gentles et al., 2010).

### Strengths and weaknesses

The study's strengths lie in its purposive sampling, which ensured participants had relevant experience and geographical diversity, and the use of semi-structured interviews supported by a robust data collection process and the PVC framework for analysis. Validating transcription accuracy with participant feedback added methodological rigour. This study has several limitations. First, the use of purposive sampling and a small sample size may limit the generalizability of findings, particularly to regions with different healthcare settings, regulatory environments, or population characteristics. The limited number of participants may also constrain thematic saturation and depth. Second, variability in interview duration – ranging from 4 to 19 min – suggests potential differences in the richness of responses, which may affect the comprehensiveness of the data collected. Third, although interviews were conducted in Albanian and carefully translated into English, there is a possibility of misinterpretation or loss of nuance during translation, despite efforts to preserve meaning and context.

### Further research

Future research should examine how medication standardisation policies impact medicine quality and variety and explore the effects of pricing and reimbursement issues on access to pediatric medications. Additionally, it would be valuable to investigate the influence of supply chain disruptions and healthcare system integration on medication availability and adherence. Understanding parents’ experiences and incorporating the perspectives of other stakeholders, such as healthcare providers and policymakers, could offer a more comprehensive view of the challenges in pediatric medication management. Moreover, addressing the perception that patent medicines are superior to generics is crucial, as this belief may affect medication choice and accessibility. Clinically, these insights could guide improvements in pediatric care by identifying key factors affecting medication efficacy and availability. From a policy perspective, this research may highlight the need for revised medication standardisation practices, more effective reimbursement frameworks, and strategies to mitigate supply chain disruptions, ultimately leading to better medication management and improved health outcomes for children.

## Conclusion

This first study on pediatric medication management in Albania underscores significant issues with access to pediatric medicines, including challenges related to accurate dosing, shortages, and pricing. To address these problems, policymakers should prioritise expanding the reimbursement list for pediatric medicines to ensure broader access and affordability. Additionally, pharmacists, who are uniquely positioned to witness the difficulties faced by both doctors and parents regarding pediatric medications, should advocate for improved access and availability. By leveraging these insights, the findings of this research can guide the development of targeted policies and health system strengthening initiatives, ultimately aiming to improve access to and quality of pediatric medicines in Albania.

## Supplementary Material

Supplemental Material

## Data Availability

The data that support the findings of this study are not publicly available. The data collected for this study are available upon request from the corresponding author, contingent upon compliance with ethical and privacy regulations. Access will be granted following approval of a data sharing agreement and confirmation of adherence to ethical guidelines.
