# Supplementary material for: ‘Challenges in pediatric medicine access in Albania: insights from community Pharmacists’
Source: J Pharm Policy Pract. 2025 Jul 2;18(1):2521425. doi: 10.1080/20523211.2025.2521425 (PMC12224724; doi:10.1080/20523211.2025.2521425)
Supplement: Supplemental Material [file JPPP_A_2521425_SM9190.docx]

**Supplemental Material**

**Appendix 1**

**Interview Questions:**

Introductions:

1. Please tell me a little about yourself.

- ***Probe:*** Years of experience; age; gender

Experience with dispensing medicines for children:

1. I am interested in looking at access to medicines for children. What is your opinion of the medicines available in Albania for use/prescribing for children?

- ***Probe:*** List of medicines on the National Health Insurance List

1. What is your pharmacy’s experience of dispensing medicines for children?

- ***Probe:*** Difficulty in getting access to medicines/shortages of supply, or not registered, or too expensive: all medicines or certainones? If certain only, which ones are the most problematic

1. In cases where the medication described in the prescription is not available what steps are taken.

- ***Probe:*** Does the pharmacist have to provide another medicine or a different medicine when the prescribed medicine for the child is not available.

1. Please explain whether the replacement medicine requires you to contact the doctor to approve the replacement? Also do you have experiences with having to look for a medicine from the same therapeutic class or a different medicine altogether?

- ***Probe:*** Does the dosage remain the same or is it with a different dosage, requiring a calculation step

1. Do you think that there are differences in accessibility for pediatric medicines versus medicines for adults in general? Why do you think so? Why do you not think so?

Prescribing in accordance with guidelines:

1. What do you understand by adherence and non-adherence to clinical guidelines?
2. In terms of you experience, are medicines that are prescribed for children according to specific approved clinical protocols and guidelines? Please tell us why you think this is so.

- ***Probe:*** - non-implementation of the protocols and instructions
- ***Probe:*** any communication with the patient/doctor regarding non-adherence to protocols
- ***Probe:*** off label use of medication and therefore a valid prescription

1. Are you able to share an experience or story about difficulties in complying with clinical guidelines for pediatric medicines?
2. - Do you have access to clinical protocols and guidelines? Are they easily accessible? What are the protocols or guidelines that you have access to and use?

Financial aspects

1. Based on your experience, do you feel that there a difference in price regarding medicines for pediatric age groups and medicines for adults? Why do you think this is so?
2. -Do you think that the current price of children’s medicines makes a different to parents and guardians in terms of getting this medicine? Why do you think so?
3. -Based on your experience, are children's medicines in the pharmacy mainly patents or generic medicines?

Impact of access to medicines:

1. In your opinion, does the access to pediatric drugs affect the adherence of children's medications?
2. So, in cases where one medication has to be replaced with another due to its lack in the market, based on your experience with patients, does this affect adherence to the medication? positively or negatively. Provide some examples please.

Is there anything else you would like to share about access to medicines for children

**Appendix 2:** Pharmaceutical Value Chain Framework (themes and sub themes levels)

**Table S1** Framework themes and sub themes levels

| **Theme** | **Sub theme-Level 1** | **Sub theme-Level 2** | **Sub theme-Level 3** |
| --- | --- | --- | --- |
| *Policy and legislation* | Policy and legislative enviroment | Government Policies and Regulations |  |
| *Medicine regulation* | Regulatory efficiency | Regulatory and Market Liberalization Issues |  |
|  | Product registered |  |  |
| *Public financing and pricing* | Medicine prices | Pediatric prices vs adult prices | Similar prices for adults and children |
|  |  |  | Pediatric medicines tend to be more expensive |
|  |  | Impact of high prices on accessibility | High prices influence treatment choices |
|  |  |  | Parents sacrifice for children |
|  |  |  | Decision making based on cost |
|  | Pricing mechanisms | Impact on pricing policies | Cost of supplements |
|  |  | Quality concers and access to brands | Impact on Quality and Access to Brands |
| *Selection* | Standard treatment guidelines | Accessibility of STG | Lack of Access to Treatment Guidelines |
|  |  |  | Uncertainty about Treatment Guidelines |
|  |  | Impact of lack of clinical guidelines | Reliance on Experience and Informal Knowledge |
|  |  |  | Knowledge Gained from Practical Experience |
|  |  | Guidelines adherence | Adherence to Doctor's Instructions |
|  |  |  | Challenges in Implementing Guidelines |
|  |  |  | Consultation with Doctors for Clarification |
|  |  |  | Variation in Protocol Adherence |
| *Reimbursement* | Coverage | Observations on Reimbursed Drugs |  |
|  |  | Coverage and Availability of Pediatric Drugs in theist list of reimbursed drugs in Albania |  |
|  |  | Limitations and Deficiencies in Reimbursement |  |
|  | Regulation of insurance schemes | Request for Improvement in Reimbursement Policies |  |
| *Procurement and supply* | Availability of medicines | General Availability and Types of Medicines |  |
|  |  | Supply Issues and Shortages |  |
|  |  | Comparison Between Adult and Pediatric Medications |  |
|  |  | Availability of Prescribed drugs and Use Practices |  |
|  |  | Impact of non availability of medicines | Patient preference and replacement of the medicines |
|  |  |  | Medicines market challenges |
|  |  |  | Adjustment and solutions to non available medicines |
|  |  | Supply impacting rational usage of medicines |  |
|  |  | Withdrawal from market | Market Dynamics and Product Availability |
|  |  |  | Impact of withrdrawal on Patient Care and Treatment |
|  |  |  | Regulatory and Market Liberalization Issues |
| Healthcare delivery | Referral | Use of referral systems | Lack of implementation of referral system |
|  | Organization of services | Integration and Coordination of Healthcare Services | Impact of medicines efficacy not known by the pharmacist |
|  | Prescribing | Prescription Generation Process |  |
|  |  | Use of Trade Names vs. Generic Names in the prescription |  |
| Dispensing | Preparing and administering | Historical Practices and Changes in Pharmacy |  |
|  |  | Pharmacists' Responsibilities and Protocols |  |
|  |  | Collaboration with Healthcare Providers |  |
|  | Pharmacist as healthcare provider/Role of pharmacist | Pharmacist's Role in Medication Management | Quality and Pricing of Medications |
|  |  |  | Communication with Doctors and Patients |
|  |  |  | Handling Medication Shortages and Substitutions |
|  |  |  | Responsibilities and Limitations |
|  |  |  | Use of Trade Names vs. Generic Names in the prescription |
|  | Limited off label use |  |  |
| *Use* | Access to medicines | Same access to medicines for children and adults |  |
|  |  | Different access to medicines for children and adults |  |
|  |  | Access issues | General Shortages in the Market |
|  |  |  | Differences in Access Between Children and Adults |
|  |  |  | Specific Medication Shortages |
|  | Adhering and defaulting | Patient's non adherence to prescriptions |  |
|  |  | Impact of non- availability of medicines on adherence | Impact of Medication Substitution or Availability |
|  |  |  | Concerns about Antibiotic Use and Adherence |
|  |  |  | Role of Healthcare Providers in Adherence |
|  |  |  | Patient Preferences and Compliance |
|  | Patient education | Challenges in Patient Understanding and Acceptance |  |
|  |  | Patient Trust and Reliability in Pharmacy Services |  |
|  | Rational use of medicines | Communication of healthcare providers in the right execution of the prescription | Communication Between Pharmacists and Healthcare Providers |
|  |  |  | Adherence to Prescriptions and Dose Adjustments |
|  |  |  | Decision-Making in Medication Substitutions |

**Appendix 3**

**Table S2: Participants characteristics**

| Characteristics | Options | n | % |
| --- | --- | --- | --- |
| Age group | 25-34 | 5 | 27.8% |
|  | 35-44 | 7 | 38.9% |
|  | 45-54 | 3 | 16.7% |
|  | >55 | 3 | 16.7% |
| Gender | Male | 4 | 22.2% |
|  | Female | 14 | 77.8% |
| Years of experience | 1-9 | 3 | 16.7% |
|  | 10-19 | 8 | 44.4% |
|  | 20-29 | 3 | 16.7% |
|  | >30 | 4 | 22.2% |

**Appendix 4:** Selected quotes for identified barriers and facilitators

**Table S3** Selected pharmacists’ quotes

| ***Policy and legislation*** |
| --- |
| *Policy and legislative enviroment* |
| *Government Policies and Regulations* |
| - "I think that the difference of the active principles molecules and supplements happened by the decision of the Council of Ministers, because they requested the unification with the prices of the region, and the active principles were already referring to the prices of the region." (R15) |
| - "For this aspect, I have a discussion, because this leads to a decrease in quality and at the same time limits the arrival of brands." (R15) |
| *Medicine regulation* |
| *Regulatory efficiency* |
| *Regulatory and Market Liberalization Issues* |
| - "I try to keep everything. I try to keep the drugs that circulate and are in the pharmaceutical yearbook of the Republic of Albania. They try to respond to the brand that the doctor wants." (R18) |
| - "The liberalization of the market caused the work in pharmacies to decline, to withdraw more drugs than necessary, and we cannot give an exact statistic of what needs to change. But the market is currently lower in pediatric drugs than the previous consumption." (R18) |
| *Product registered* |
| -“There are also drugs that are not registered, there are even essential drugs that are not registered that are missing from the market. Some have been and are no longer, some have never been”. (R3) |
| *Public financing and pricing* |
| *Medicine prices* |
| *Variation in Price Between Adult and Pediatric Medicines* |
| *Similar Prices* |
| - "The price is the same, there is no change." (R5) |
| - "It is the same for adults, it is not that there is any difference between children and adults in this part." (R9) |
| Pediatric Medicines Tend to be More Expensive |
| - "Medicines tend to be cheap. The ones that are patent are mostly at expensive prices, despite the fact that there are many shortages of patent medicines in Albania." (R1) |
| - "Yes, there is a difference. In general, pediatric drugs tend to be priced slightly higher than adult drugs." (R14) |
| - "Yes, yes, it depends, I said for children there are fewer alternatives on the market, but in general, the trend is for them to be more expensive." (R3) |
| - "Amoxicillin syrup for children is missing, with which the right. But if we refer to any other therapeutic class or other therapeutic drug, there is a difference. Prices for children are slightly more expensive than for adults." (R17) |
| Impact of high price on accessibility |
| *Financial Burden on Parents* |
| *High Prices Influence Treatment Choices* |
| - "It definitely affects, being the high price, it definitely affects. They think twice, make compromises to take something and leave something else from the recipe." (R4) |
| - "Yes, it affects. There are even cases where they tell us to find something cheaper, so they can change the active principle, just so that they can afford to buy that type of antibiotic for their children." (R7) |
| - "Some of them do not even have the opportunity, because the refund does not work, to receive a refund." (R12) |
| - "There are medicines that they cannot take because they are more expensive than they can afford." (R13) |
| - "It is having an effect, there are medicines that they cannot take because they are more expensive than they can afford." (R17) |
|  |
| *Parental Sacrifice and Affordability* |
| *Parents Sacrifice for Children* |
| - "No, generally parents sacrifice for the child and price is not a controversial part for them." (R5) |
| - "Very few cases when they make a calculation of the prescription than it goes to them, we always talk about the economic point of view." (R15) |
| - "They can't even afford it." (R18) |
|  |
| Decision-Making Based on Cost |
| - "Yes, there are certain classes that can't afford all of all the prizes and start with then say who is the most urgent to get, who can I leave for now." (R2) |
| - "For the same active principle, there are several companies that offer different prices, if the parent does not have the opportunity to get the drug at higher prices, he can get his alternative, with the same dose, with the same doctor's prescription, in his financial opportunity at a lower price." (R8) |
|  |
|  |
| Pricing mechanisms |
| Impact of Pricing Policies |
| Cost of Supplements |
| - "One thing that is noted to be expensive for both pediatric age and adults are supplements." (R15) |
| Quality Concerns and Access to Brands |
| *Impact on Quality and Access to Brands* |
| - "The brand is something that the Albanian population should have the right to enjoy, according to everyone's opportunity." (R15) |
| - "It is understood that generics were born as the most economical alternative for the wider population, but not to take away the right of the brand to be and the citizen who will be treated or treated in this case with a medicine, what is the brand." (R15) |
| **Selection** |
| Standard treatment guidelines |
| *Accessibility of Treatment Guidelines:* |
| - "Yes, they are easily accessible." (R7) |
| - "Yes, they are accessible. Even in those cases where the way of writing is not regular, enter these protocols to find it." (R10) |
| - "In general, yes, they are understandable and more than easily accessible. We have not encountered any difficulties in this aspect." (R9) |
|  |
| Lack of Access to Treatment Guidelines: |
| - "Where will we look, where is the protocol, we have not had information about this, it has been years." (R12) |
| - "Unfortunately, I wanted to say that we do not have much access to approved clinical protocols and on this point to be honest, considering that we do not have much information on this type of profile, despite private efforts, we cannot judge what the doctor prescribes." (R17) |
| - "I do not have the access to neither detect nor have a collaboration or ask, apart from ethics, legal right and apart from ethics or the human one that we can communicate, because it seems as if we are prejudicing the doctor." (R15) |
|  |
| *Uncertainty about Treatment Guidelines:* |
| - "It is not that we have written things that have come to us exactly, that is, to have them as a protocol, but they are unwritten things that we know more or less." (R2) |
| -“We, the pharmacy, do not have access to protocols. The protocols are accessible to the doctor, they are those who follow the protocols and other institutions, those who follow the doctor's protocols. We have no right to interfere with the protocol”. (R4) |
| **Impact of lack of clinical guidelines** |
| *Reliance on Experience and Informal Knowledge:* |
| - "No, we don't have any [protocols], but the drug lists are written according to the protocol and we learn from experience."R18 |
| - "From the occasional contact with the doctor, because we have to keep contact for the sake of work, we understand that there are limitations plus we know them ourselves." R9 |
| - "Most of the pharmaceutical protocols and incompatibilities we learned in school." R18 |
| *Knowledge Gained from Practical Experience* |
| - "We know very well that a Quinolone preparation, for example Fluoroquinolone, is not used in pediatrics." R18 |
| - "Despite the fact that one pharmacopoeia is up to fourteen years old, another may be up to eighteen years old, we again try to correct the doctor, if necessary." R18 |
|  |
| Guidelines adherence |
| *Adherence to Doctor's Instructions* |
| - "Then compliance with the instructions. The idea is that we, if it is about the prescription, that is, compliance with the dosages, the time of administration, we call compliance with the instructions given by the doctor. We execute, we do not give the prescription ourselves from the beginning, so we normally stick to what the doctor prescribed." (R2) |
| - "Compliance with the prescription, based on the medication prescribed by the doctor, is generally respected. In cases where we have no doubt, we communicate with the doctor and resolve it." (R6) |
| - "Then, we try based on the prescription that comes from the doctor, we always try to stick to the prescription and protocol that the doctor has set." (R14) |
|  |
| *Challenges in Implementing Guidelines* |
| - "It is not always welcome, that is, the idea is that you have doctors who are very cooperative, but there are also doctors who are not or are not available or are not very happy to approach the opinion that we have versus what is written." (R2) |
|  |
| *Consultation with Doctors for Clarification* |
| - "In the first case, I conduct a more extended interview with the child's parent and in case I really think that there is something wrong, he must definitely communicate with the doctor, that is, I refuse to execute the prescription without talking to the doctor. If the doctor then insists that it is ok and has considered the choice he has made, fine. But I have to verify it." (R3) |
| - "Yes, we even had contact with the doctor, who then removed it.Thus in the case of an Amikacin for a young child that is not used, or the case of Ciprofloxacin for children under fourteen years old that is not used. In these cases, we contacted the doctor." (R12) |
|  |
| *Variation in Protocol Adherence* |
| - "At least with these oral cortisones, the protocol is not always followed exactly as explained. There are also doses that are not given in the same way as explained." (R2) |
| - "I don't mind, because according to the protocols, it is thought that the child can take the antibiotic three or four days from the onset of the fever, but there are cases that he takes it even on the first day." (R12) |
| Reimbursement |
| *Coverage and Availability of Pediatric Drugs in the AMHIF list* |
| - "For pediatric drugs, the range should be wider, there is much to add. That is, the list of drugs should have been wider. Some should be removed, some should be added. There is a lack of cytotoxic, drugs for children with leukemia, they are not on the pharmaceutical market, maybe they are in hospitals, but they are missing on the market. There is always room for improvement. Pediatrics is very nice and very caring. We must be very careful when giving pediatric drugs." (R18) |
| - "In Albania, the list of reimbursable drugs does not meet all the requirements of children and parents for children. It is understood that they are then covered outside the list, while in the pharmacy they are all [available]." (R16) |
| - "The range of pediatric medications included in the reimbursable list of drugs is normally very low, in relation to the demands that the Albanian pharmaceutical market may have." (R17) |
|  |
| *Limitations and Deficiencies in Reimbursement* |
| - "There are restrictions on the reimbursement part as far as the pediatric sector is concerned, unlike the other part. There is a limitation in diagnoses and accessible drugs, in the reimbursement part." (R3) |
| - "The daily practice is that the molecules with which pediatricians treat pediatric patients are generations of Cephalosporins, whether in injections, which are not on the reimbursement list for any category, even in suspensions, that is, syrups such as the antibiotic Azithromycin, Cefradur, Cefixime. As molecules, without mentioning them as trade names, Cefprozil. These are mainly the range of things that pediatricians, pediatricians, apply. So the benefit in a nutshell, if that's your question, that the pediatric patient benefit from the reimbursement list is very very minimalistic, almost zero." (R15) |
| *Observations on Reimbursed Drugs* |
| - "Refer to the list of reimbursed drugs. Currently, there are a variety of companies in Albania, including Albanian ones, but there are also many patent brands that offer medicines for pediatric use. It means referring to the list of reimbursable drugs. We have, that is, there are many drugs in the drug list." (R1) |
| - "In the list of reimbursable drugs, we have almost 70% of the drugs." (R5) |
| - "Thus, with the part of the Fund, that is, the reimbursement, it is not that I have worked, I do not know this part of reimbursable drugs. Here we have it without refund." (R4) |
| - "There are absolutely no prescriptions with refunds, absolutely, once before there were, while today they are all R3 prescriptions." (R12) |
| *Regulation of insurance schemes* |
| *Request for Improvement in Reimbursement Policies* |
| - "It is one thing that we should ask the legislative and law-enforcement bodies for the reimbursement list to be richer, with pediatric medications." (R15) |
| - "Prescribed pediatric medicines not covered in the AMHIF list. For the part of the health insurance list, at least in our area there are deficiencies, I see that prescriptions for children are only white* empty, so currently there are prescriptions with R3 which are paid. I can have very little through the list that can come to me from doctors or prescriptions with reimbursement." (R7) |
| Procurement and supply |
|  |
| Availability of medicines |
| *General Availability and Types of Medicines* |
| - "They are generally generic. Rarely can there be a patent bar, but most of the ones we give out during the day are all generic." (R7) |
| - "Now there are both patent medicines and generic medicines." (R10) |
| - "We have patents and generics, but who has a patent left? Maybe Agumentina syrup, maybe some Azithromycin Sandoz." (R18) |
| - "Rather than patents, there are also generics such as Clamox or Betamox." (R9) |
| - "Medicines that are registered, that are registered by the state, we have had them at all times, even if the patient definitely wants to take it, we order it and give it to him, unless he takes it in the morning or in the afternoon." (R10) |
| - "The prescription comes from the pediatrician, we have medicines for children and we distribute them regularly." (R11) |
|  |
| *Supply Issues and Shortages* |
| - "At certain moments and certain periods when there is some flow, it happens that there are such problems that there are shortages in the market. Medicines then, for the price part, are diverse, i.e. of all types, starting from the lowest prices, which are generally domestically produced, to the slightly higher prices from imports." (R14) |
| - "From time to time, at different times, there are shortages in the market." (R3) |
| - "As we said, there are shortages." (R2) |
|  |
| Comparison Between Adult and Pediatric Medications |
| - "I think that adult drugs have a wider range of both use and access." (R3) |
| - "There are more drugs for adults than for children." (R5) |
| - "Now I don't know what to tell you more, because adults are more consumers considering that the adult age group itself starts from fifteen or seventeen years old and up to the old age of eighty or one hundred years old and they are the chronically ill mainly, that they have hypertension, diabetes, coagulation and other respiratory problems. So, in general, the biggest consumers and according to the typology that I have as a pharmacy are them." (R15) |
| - "There are generally more drugs for adults. Years ago, the population was larger and there were many medicines for children. Now there are fewer children and fewer drugs." (R10) |
| - "For use, they are used more for adults, definitely, because the number of the population in the third ages makes the use in the third ages to be much greater." (R12) |
|  |
| *Availability of Prescribed medicines and Use Practices* |
| - "They are generally taken by prescription, very few can be OTC which we also advise ourselves that we give cases either for temperature or for pain such as analgesics. The rest can be prescribed in the form of a prescription, that is, always from the doctor, because it happens that children can have different allergies and definitely not every medicine or antibiotic can be used." (R7) |
| - "At the moment they are all complete, at least the medicines that are the main ones for the child are totally accessible." (R7) |
| - "Our experience of medication, that is, the source of pediatric medication is the prescription issued by the pediatrician, who are the pediatricians of the health centers, even pediatric of the regional hospital of Vlora, its pediatric department. The recipes they generate in them are mostly findable, not impossible." (R15) |
| - "Look, the replacement situations in my memory is not that I remember for two reasons. Maybe not because it didn't happen and it could have happened after consulting with the doctor, but because of the fact that when I keep the range of medicines, even the quantity in the range, in the type, it is that this element in me is not able to precipitate in this situation." (R15) |
| \| *Impact of non-availability of medicines* \| \| --- \| |
|  |
| *Patient Preference and Replacements* |
| - "The idea is that patients don't like replacements. That is, the first thing they say is give me what the doctor gave. That is, if there are shortages, we are the ones who take them to the doctor to see how the replacement can be done." (R2) |
| - "In many cases it requires a doctor's approval. Although in some other cases it can be done with the consent of the patient. That is, clarifying or talking to the patient, explaining the equivalent that exists in the market of that medicine, which is probably in short supply and what was prescribed by the doctor is not available. Then agree to replace it with a similar equivalent." (R4) |
| - "Definitely yes. It happened, it happened recently. An antiparasitic that has been absent from the market for a long time, definitely affects and is replaced and is not the right one and does not work." (R4) |
|  |
| *Market Challenges and Atypical Situations* |
| - "The market here is a bit atypical, that is, here there are all three of these problems that you mentioned, all three can happen or can be encountered during the work of all three. The doctor, for example, in one case describes a drug that is not available on the market here, it is not accessible, because he knows that type of drug exists, but it is not here. And people ran around to find it, try and come to it then, find the equivalent or something similar. I mean, here is the everyday, the three things you mentioned above happen." (R4) |
| - "There are preparations that are not found in Albania, such as various syrups. But our pharmacy can also prepare them as galenic preparations." (R5) |
|  |
| Adjustments and Solutions to non-available medicines |
| - "But the current shortage in the market is not that there is anything that could be missing, maybe by adjusting the doses we can make it possible for the child to take it. Even if the dose is missing, we adjust it by adjusting the dose." (R7) |
| - "When it is not present, with the patient's wish, I can propose that to be ordered and the patient receives the order all afternoon. Otherwise, I return the prescription to the patient." (R10) |
| - "We can contact the doctor or wait for the next supply that comes within the day, at a time when we don't have it and the patient comes and gets it in two hours, because the supply can be very fast." (R12) |
|  |
| *Supply impacting rational use of medicines* |
| There are shortages, at least of what I call antibiotics, amoxicillin in this case, which starts with slightly more advanced classes, that is, with the third class of antibiotics or the fourth, without going to this first. R2 |
| In those pediatric prescriptions we receive, however we are not happy that the third generation active principle is written, in a small child as young as six months. R12 |
|  |
| *Withdrawal from market* |
|  |
| *Market Dynamics and Product Availability* |
| - "Here the problem is a bit higher, in the broader aspect of the market. There are problems here that some drugs are introduced and some others are not, I don't know what the policies are. Other drugs are in short supply for a long time, disappear especially some international patents that disappear and it is not understood why and some other second-hand ones are preferred." (R4) |
| - "No, at this time we are talking, for example, the Ventolin pump that is for Asthma, not only Ventolin but also others that replace Vettolina, are missing. For example, Salbutamol is missing, Madopari has been missing for two months." (R10) |
| - "As far as the medicines available in Albania are concerned, in the past the range of medicines for children was more extensive, while now there are many shortages." (R9) |
| - "I am concerned by the fact that we have almost no patents at all in our pharmaceutical market, especially in the pediatric part. There have been shortages from time to time, which are amortized by the equivalents of the same dosage form, the same class." (R17) |
|  |
| *Impact on Patient Care and Treatment* |
| - "Yes, there were periods of time when there were quite a few shortages, but then we were able to, since we are a large pharmacy, at least for the area, for over twenty years in the market as an operation, we have tried based on the conditions set by pharmaceutical warehouses, we have tried to fill at least what our patients have been, let alone the shortages that have been in the market." (R7) |
| - "In general, we are faced with a shortage. There is no problem with the expensiveness, because in general parents do not have a problem spending on children, especially when it is medicine for fever or an infection, but there are frequent shortages that cause problems in the treatment of children." (R6) |
|  |
| Regulatory and Market Liberalization Issues |
| - "I try to keep everything. I try to keep the drugs that circulate and are in the pharmaceutical yearbook of the Republic of Albania. They try to respond to the brand that the doctor wants." (R18) |
| Healthcare delivery |
| Referral |
| Lack of implementation of referral system for the pediatric prescriptions |
| Use for reimbursable There is something in Albania, a parent tends to go directly to the pediatric specialist, bypassing (R1) |
| But, for children or parents who have an acute or chronic problem, but mostly acute, they don't go to the doctor, they don't follow the route of referrals but go directly to the specialist, so we bypass the reimbursement in this way, while those who have chronic problems , they do They normally apply to it, whether for diabetes problems, for problems with diabetes or metabolic problems They are implementing it, despite the fact that in the case of the metabolic problem there are two or three missing from the list of drugs. (R1) |
| **Organization of services** |
| *Integration and Coordination of Healthcare Services* |
| "But the fact that I follow this thing, we should have a very connected system. If we were in the system, it would be known that the child is visited in the pediatric hospital of the city of Vlora, the drugs are taken in this pharmacy. It is a closed cycle not in financial terms, but a closed cycle of service control and again in re-control, and then we can investigate and have a clearer picture of their efficiency." (R15) |
| *Prescribing* |
| Prescription Generation Process |
| - "To generate a prescription, in principle, the doctor must be a specialist or maybe even a general practitioner, but the generation requires the implementation of several types of procedures." (R1) |
| - "The prescription that has been generated and we cannot prejudge whether or not the preliminary protocol that generated this prescription has been implemented, we can make a judgment on the prescription, whether or not there is suitability of Medicines, one with the other, on the dose, whether or not there is suitability of dose for the pediatric age, in the event that we do not have the opportunity with this opportunity, we have to judge whether it is This has been implemented previously directly from the doctor Protocol to find prescription routes." (R1) |
| *Use of Trade Names vs. Generic Names* |
| - "Usually we have prescriptions that are written by trade names, we do not receive prescriptions that are named after the chemical component so that we can choose depending on the population, the economy of the patient or many other factors. But they come to us written with commercial names, that is, according to an R3 prescription so that the doctor determines what the patient, a child in this case, will receive." (R7)  . |
| - "But my personal opinion is that we should work with generic names, it should be improved, there is a lot of room for improvement. Should another electronic prescription be made, such as for drugs on the reimbursement list, there is much room for improvement." (R18) |
| Dispensing |
| *Preparing and administering* |
| Historical Practices and Changes in Pharmacy |
| - "When I became a pharmacist for the first time, children's medicines were mostly prepared in pharmacies, for example Pertussin syrup, Tusipectol syrup, Tetracycline syrup, Chloramphenicol syrup were prepared in pharmacies. We had all the conditions to prepare them. We had mortars, test tubes, drums. For example, Pertussin syrup or Tusipektol syrup was prepared in a large canister, and then divided into 100g packages." (R10) |
| - "At that time, we were told that when you deal with table A, your hand will shake. Now I understand this saying, because it was heroin and cocaine. I also specifically had the license for narcotic drugs because I had two patients. I remember one of the patients was using Morphine. Only I had this right in Kavaja, but of course according to the norms. There have also been cases of threats because imagine they are drugged." (R10) |
|  |
| *Pharmacists' Responsibilities and Protocols* |
| - "So, in some cases the prescription does not specify the dose at all. Then we calculate the dose based on the child's weight. When the dose is determined, we always stick to that line, to that dose. If the dose is missing, then we contact the doctor." (R2) |
| - "Regarding the execution part, I try to be correct, because I think the doctor has a clearer understanding of the situation, even more so in pediatrics." (R3) |
| - "No, we do not replace them, because it is not even the wish of the doctor who gave the prescription. The doctor strongly insists on the brand he gave himself." (R10) |
|  |
| *Collaboration with Healthcare Providers* |
| - "I also have a history of dose inconsistencies. The dialogue has been for the better, in most cases it has been resolved. Sometimes the doctor made a lapse/unintended mistake, sometimes he didn't and I wanted to verify it." (R3) |
| - "Things we notice in the prescription may be that its elements are not all filled in order, from the head. For example, I have to ask the child's parent how old the child is, because it is not defined. I also ask about weight, because today we notice that children are also obese, and so we deviate a little from the most classic norms for determining doses, especially for antibiotics." (R15) |
|  |
| *Handling Medication Substitution and Adherence* |
| - "Yes. For example, Agumentin for children syrup, when they come with a prescription they write Agumentin, and we always give them Clavunate or Klamoxin syrup, always in the same doses." (R13) |
|  |
| *Pharmacist's Role in Medication Management* |
| *Use of Generic vs. Trade Names* |
| - "Usually we have prescriptions that are written by trade names, we do not receive prescriptions that are named after the chemical component so that we can choose depending on the population, the economy of the patient or many other factors. But they come to us written with commercial names, that is, according to an R3 prescription so that the doctor determines what the patient, a child in this case, will receive." (R7) |
| - "But my personal opinion is that we should work with generic names, it should be improved, there is a lot of room for improvement. Should another electronic prescription be made, such as for drugs on the reimbursement list, there is much room for improvement." (R18) |
|  |
| *Quality and Pricing of Medications* |
| - "I don't know how to say the rest, because I am a partisan of a medium and high category quality medicines and this is normally reflected in a price. I do not prefer the category with a low price, because I am of the principle that the quality goes with the price and as such I have made this selection so that what the patient receives is beneficial and ends with a successful recovery of the patient pediatric." (R15) |
| - "If it is another molecule, normally it is different, but since no molecule has been changed, the dose update belongs to the pharmacy. Adequacy of dosage is the responsibility of the pharmacy." (R1) |
|  |
| *Communication with Doctors and Patients* |
| - "We always give with a doctor's prescription; we prefer not to give medication except OTC drugs at least in children." (R2) |
| - "We prefer communication with the doctor, we always either have them as registered numbers, or when they have it written in the prescription, we also ask the patient, but always. In cases where they do not have the number, we prefer to return the patient to the doctor." (R2) |
| - "I prefer to contact the doctor in cases where it is not that class at all, in cases where the class is possible and has the same dose, I prefer to make the change myself, but this is for the pharmacist to change the part himself without spoiling what the doctor gave first." (R2) |
|  |
| *Handling Medication Shortages and Substitutions* |
| - "In general, yes, the correct dosage is written in the prescriptions, for example how many milliliters the suspension will be, the dose related to how many milligrams per kilogram of weight, specifying how many times the child will take it. So responsibly yes, it is according to protocol." (R9) |
| - "It is a matter of substitution within the same active principle and with the same dosage, it does not change." (R11) |
| - "When it is not present in the pharmacy, we will either give the same active principle, when talking about the generic, we give the same active principle, or then communicate with the doctor." (R16) |
|  |
| *Responsibilities and Limitations* |
| - "The pharmacist must do everything. for health, yes, without exceeding the powers given by the Pharmacist Law, that is, if it is a question of updating Or dose, or if we do not have the prescribed dose, you can take Another dose to adjust it, through the product you have in the pharmacy. But if the molecule or active principle must be changed, the doctor's opinion or decision should not come into play." (R1) |
| - "We calculate the dose ourselves." (R18) |
| - "We only have the right to prepare when they are not available on the market, when there is no other alternative on the market." (R5) |
| Limited off label use |
| -“Yes, it is not that there is much problem, what is prescribed is given” ( R2) |
| **Use** |
| Same access to medicines for adults and children |
| -“No, I find both equally accessible. I don't see access as a problem here. “ (R4) |
| Different access for children and adults |
| -“Yes, there is a difference in pediatric drugs and drugs for adults, you need to be very careful with the dose, because in children the dose calculation is based on weight.” (R8) |
| *Access issues* |
| *General Shortages in the Market* |
| - "That is, there is a shortage in the entire market, in any field. That is, there are cases that the people at the pumps, which are for adults, have been very lacking during this time, they are drugs which are vital for patients who are lacking in the market." (R2) |
| - "The problem here is a general problem of the Albanian market. There are many shortages. It is not only in medicines for children, but there are shortages in general. One moment in a period of time the drugs are found and everything seems to be going normally, then suddenly there are shortages." (R4) |
|  |
| *Differences in Access Between Children and Adults* |
| - "No, I don't think there is a factual difference. It's the same for adults, there are difficulties on both sides. Not that there is any difference. For adults it is more pronounced, so the shortage is greater, while for pediatric drugs I think it is more affordable." (R7) |
| - "For children, there may be one or two drugs that are missing, while for adults we have quite a few shortages. At ages over twelve-thirteen, or fourteen, there are quite a few shortages and have been expressed for more than a year or six months, as shortages in the market." (R7) |
|  |
| *Specific Medication Shortages* |
| - "One of the main problems currently is the breathing pump, i.e. Salbutamol which is currently in short supply." (R7) |
|  |
| *Adhering and defaulting* |
| *Patient's non adherence to prescriptions* |
| -“In cases, for example, when it is prescribed that cough syrup will be used, we are saying until the end, there are cases that stop as soon as they see an improvement, so there are cases that that advice is not followed until the end.” ( R2) |
| *Impact of non- availability of medicines on adherence* |
| *Impact of Medication Substitution or Availability* |
| - "If it is not and as a molecule Then, normally it affects, but if as a molecule there is an alternative, absolutely not." (R1) |
| - "I think there is no difference, for example it is Davixon, it is Dixon, it is Forsef. I personally would prefer Davixon, but I have seen plenty of cases that have felt good with others as well. It also depends on the budget." (R10) |
|  |
| *Concerns about Antibiotic Use and Adherence* |
| - "I would like there to be no shortages, it is the class, that is, it is the part of the children, it is what is needed both in the part of the use of drugs and in the part of access to drugs, everything should be well thought out, not giving antibiotics without criteria." (R2) |
| - "There are cases where they do not complete the treatment, as soon as they see a small improvement, they stop the treatment and do not take it to the end." (R6) |
| - "Yes, it has an effect, it must be taken according to the prescription, especially the antibiotic at the moment they start, it must be taken for five days, it must be taken for five days, no matter how well the child becomes at two days old." (R8) |
|  |
| *Role of Healthcare Providers in Adherence* |
| - "I believe that the doctor thinks well before giving a medicine to the child, the pharmacist thinks twice to see the doses, so I don't believe, we haven't had any cases." (R4) |
| - "Feedback on the progress of conditions, I cannot say that we can have regular information, because the number of patients referred to us by their parents regarding the child's progress is very small. The doctor has more access to this part. He is the one who monitors, observes the progress of the child's condition." (R17) |
|  |
| *Patient Preferences and Compliance* |
| - "It is difficult, they ask for what they are used to, but the doctor's prescription is not discussed anyway." (R11) |
| - "No, it has no impact, the patient takes and completes the medication, regardless of whether or not it is a replacement by the doctor. In general, adherence to medication is good." (R9) |
| - "Yes, we get informed that the child is doing well. I am known here and I also know the parents and often when I meet them, I ask them." (R18) |
| *Patient education* |
| *Challenges in Patient Understanding and Acceptance* |
| - "The difficulty is only in the lack of understanding from the patient, i.e. the parent of the child who often does not understand, for example, that we are on the same active principle and refuses." (R7) |
| - "In other words, he always tells you: 'If you don't have it, with this name that the doctor gave me, I won't take it.' This is one of the problems we face every day." (R7) |
|  |
| *Patient Trust and Reliability in Pharmacy Services* |
| - "But parents are patients of some pharmacies, in which they find the medicines, where they can solve their issues. This needs a deep analysis." (R15) |
| - "Anyway, at least I can say a fact that quite a few parents, even adults, come to my pharmacy and ask for advice, and this is the success of a job, reliability and trustworthiness comes on the basis of the result that has been achieved through the responsibility of giving drugs, of proper advice and dosages and quality, above all." (R15) |
| Rational use of medicines |
| *Communication Between Pharmacists and Healthcare Providers* |
| - "Mainly by phone, because it is the fastest way." (R1) |
| - "We also calculate it ourselves, but for the safety of the patient we also contact the doctor, there is always doctor-pharmacist contact." (R12) |
| - "In cases of shortages, we normally communicate with pediatricians. We are all in touch by phone with them and are available to discuss." (R15) |
| - "We contact the doctor to change it, in case you have no other alternative, you cannot give it yourself." (R8) |
| - "As I said, I don't leave this in my space of action, but I contact the doctor and he is the one who decides how we should proceed." (R9) |
| - "Of course, if I need to replace a medication, I should contact the doctor in question, the pediatrician." (R11) |
| - "Normally, we contact the doctor, if the medicine is missing in Albania, we contact the doctor and send the patient back to consult his pediatrician once more." (R17) |
|  |
| *Adherence to Prescriptions and Dose Adjustments* |
| - "Yes, if the doctor gives ok is being used. So, regardless of the fact that the prescription can be requested later as a document, but that." (R1) |
| - "We also calculate it ourselves, but for the safety of the patient we also contact the doctor." (R12) |
| - "We mainly operate, we refer to doses of the average actionable level, of course." (R15) |
| - "When there are dosage problems, we always discuss it with the doctor." (R5) |
| - "In general, it is the implementation of the prescription, rather for absences we have contact with the doctors, but the prescriptions are generally correct and we prescribe them according to the prescription." (R15) |
|  |
| *Decision-Making in Medication Substitutions* |
| - "For antibiotics, without the doctor's opinion, we cannot change it. While we can change a cough syrup, for example, syrup for a cough with secretions or for a dry cough, which are available in pharmacies and without a prescription." (R12) |
| - "I had a situation regarding the use of Cefaclor, for a two-year-old child, that I thought the dose given by the doctor was too much, and I normally take it on the phone and call it his slip." (R15) |
| - "I don't leave this in my space of action, but I contact the doctor and he is the one who decides how we should proceed." (R9) |
